# Supplementary figures and images for: Structure of dual BON-domain protein DolP identifies phospholipid binding as a new mechanism for protein localisation
Source: eLife. 2020 Dec 14;9:e62614. doi: 10.7554/eLife.62614 (PMC7806268; doi:10.7554/eLife.62614)

## Slide 1
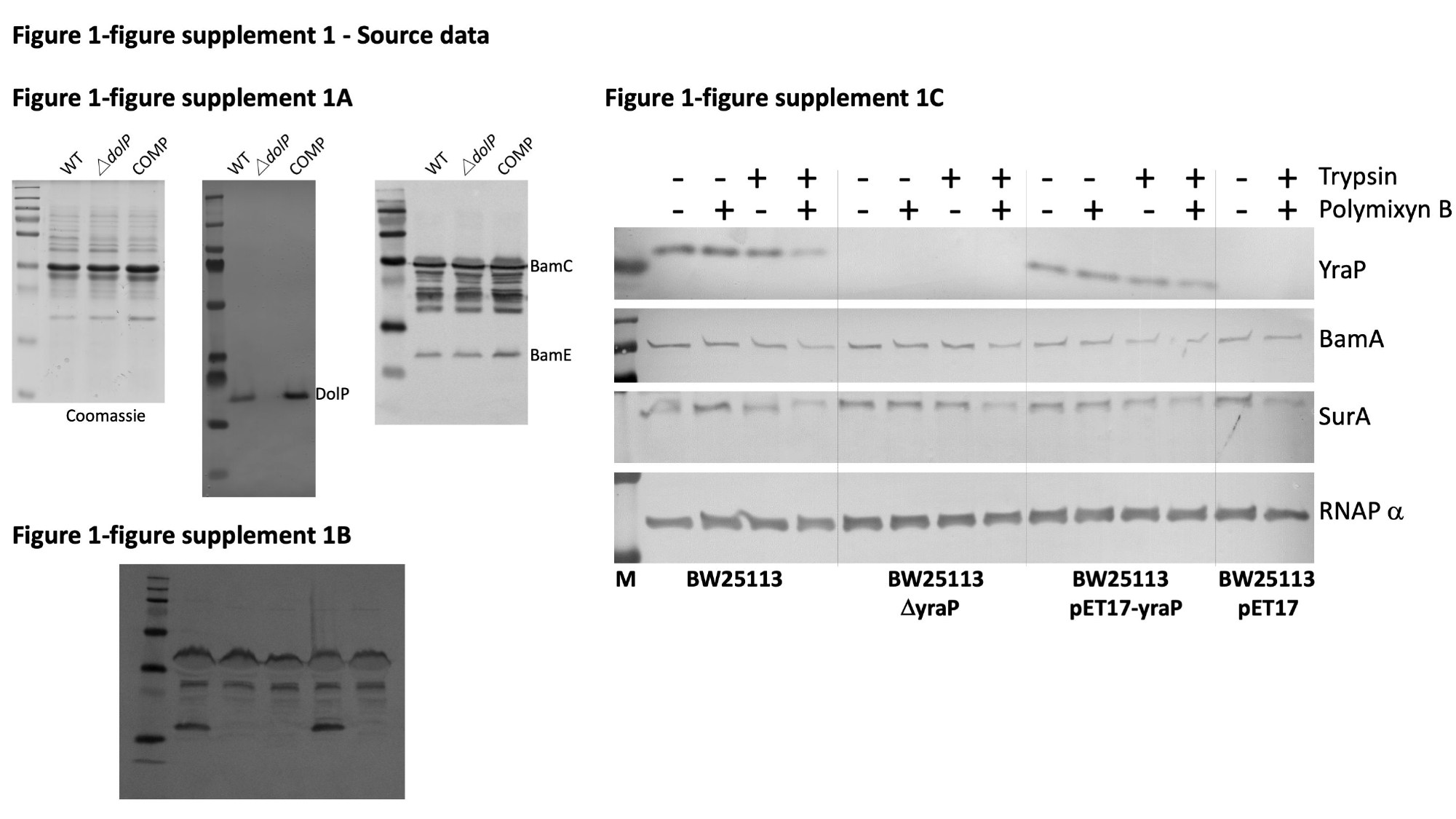

Supplement: Figure 1—figure supplement 1—source data 1. [file elife-62614-fig1-figsupp1-data1.pptx]

## Slide 1
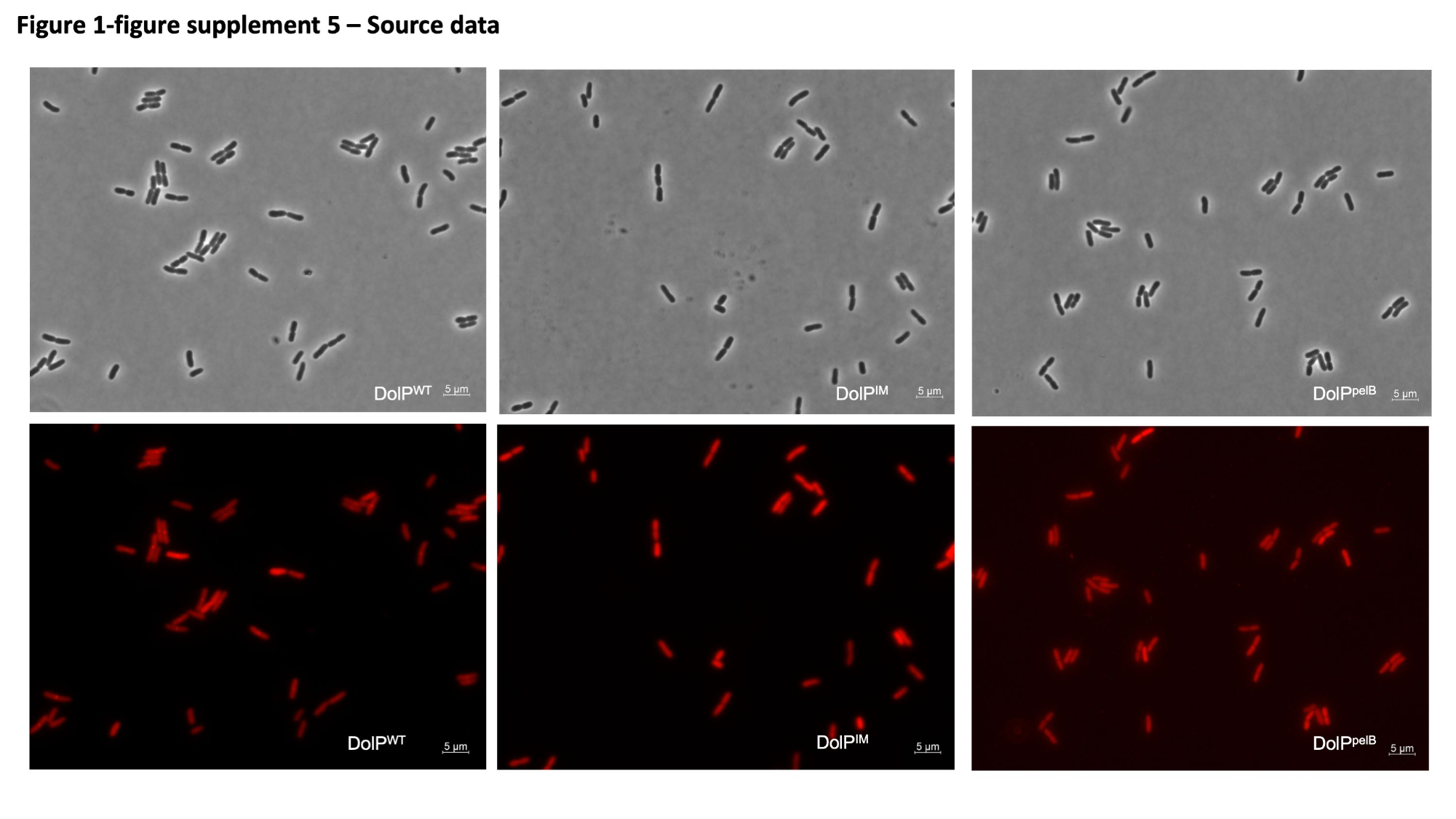

Supplement: Figure 1—figure supplement 5—source data 1. [file elife-62614-fig1-figsupp5-data1.pptx]

## Slide 1
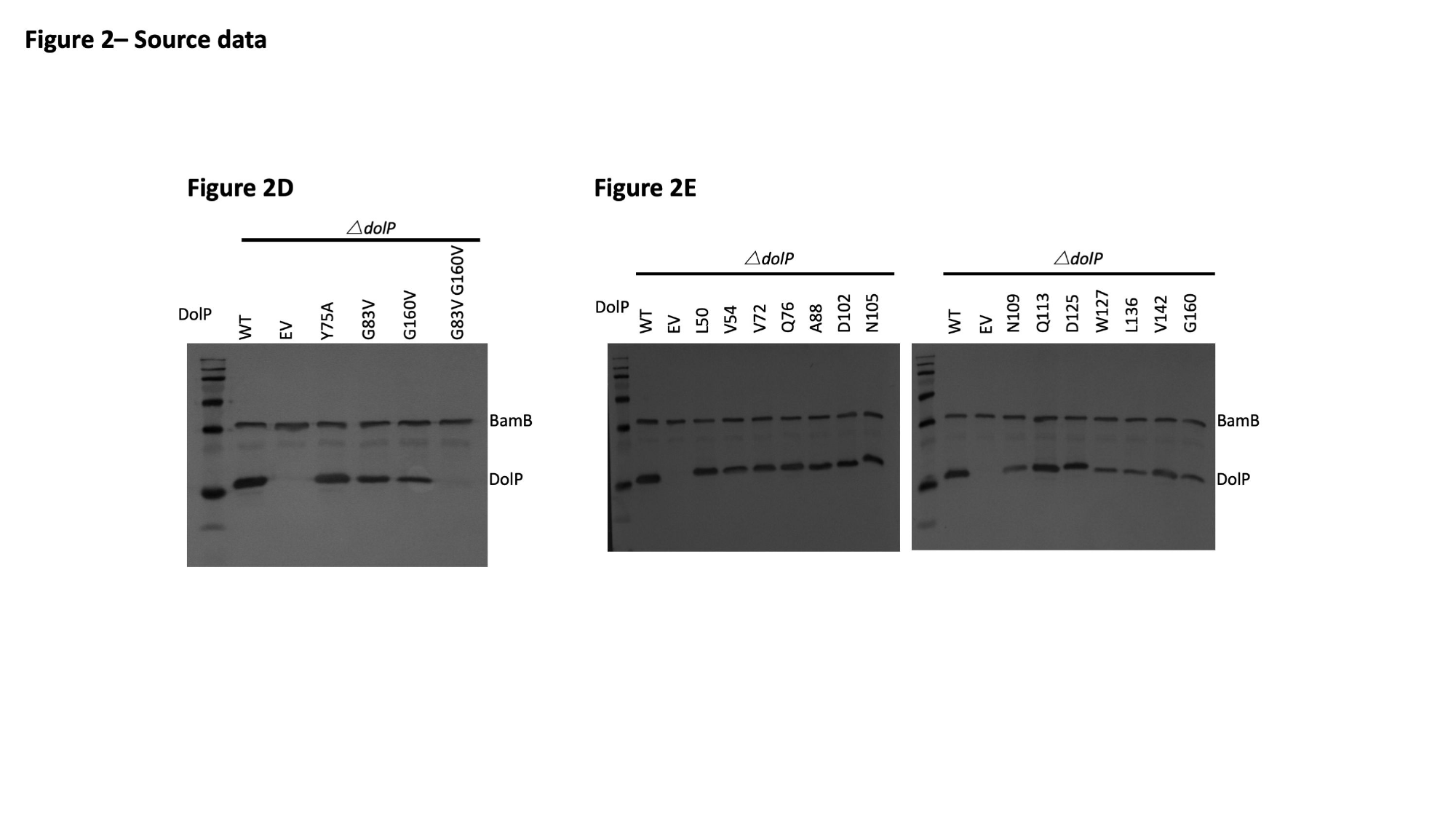

Supplement: Figure 2—source data 1. [file elife-62614-fig2-data1.pptx]

## Slide 1
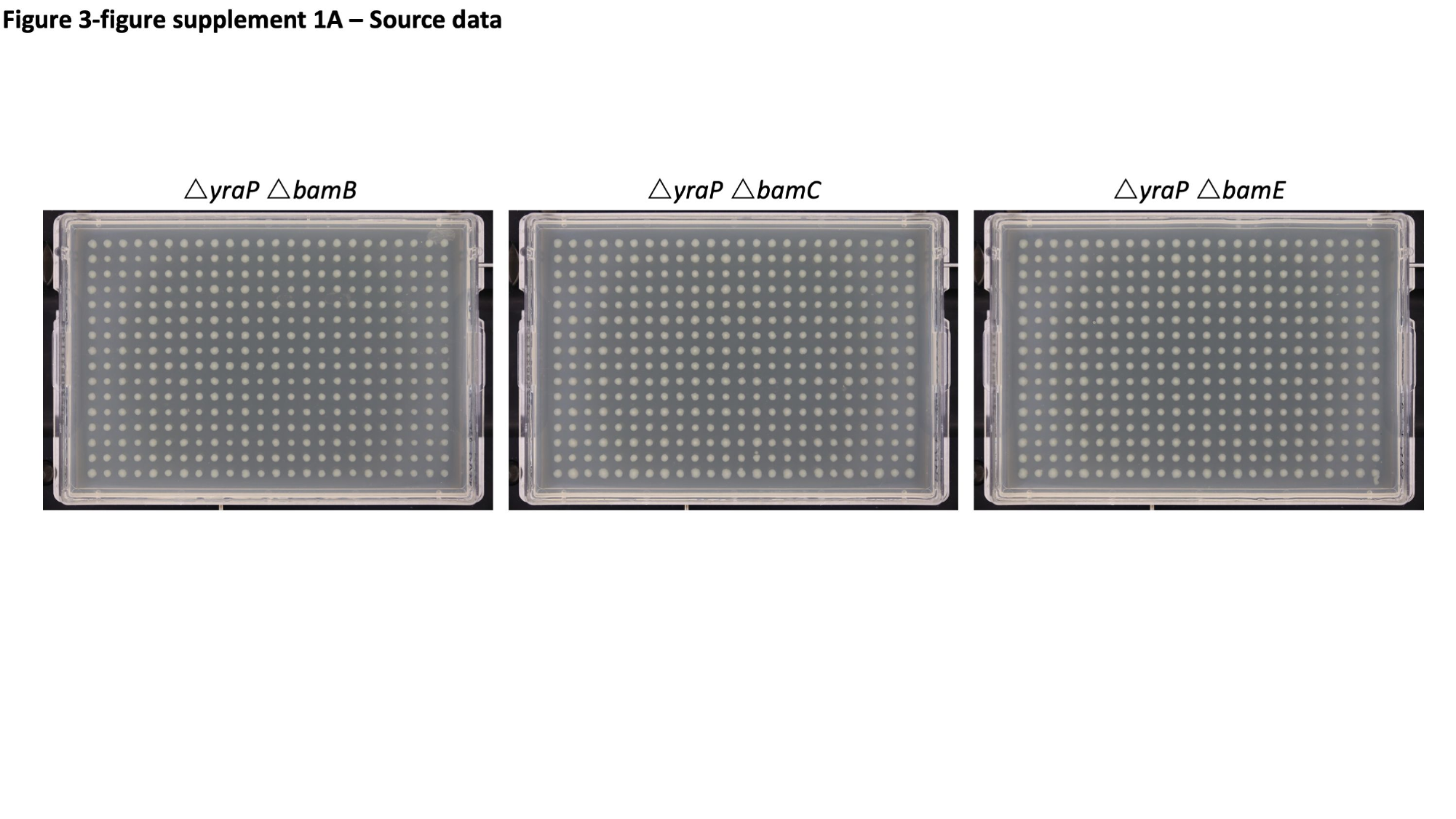

## Slide 2
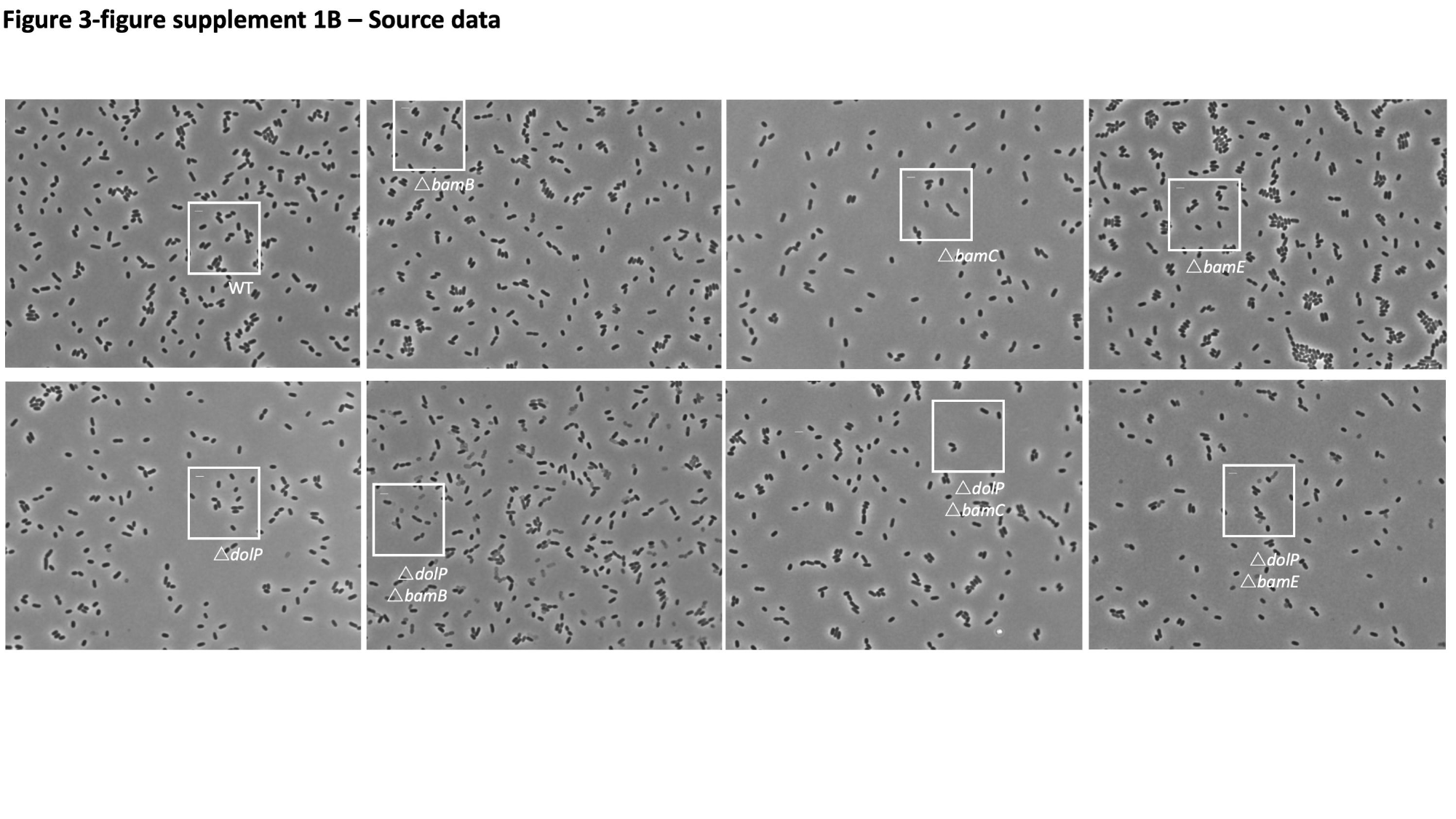

Supplement: Figure 3—figure supplement 1—source data 1. [file elife-62614-fig3-figsupp1-data1.pptx]

## Slide 1
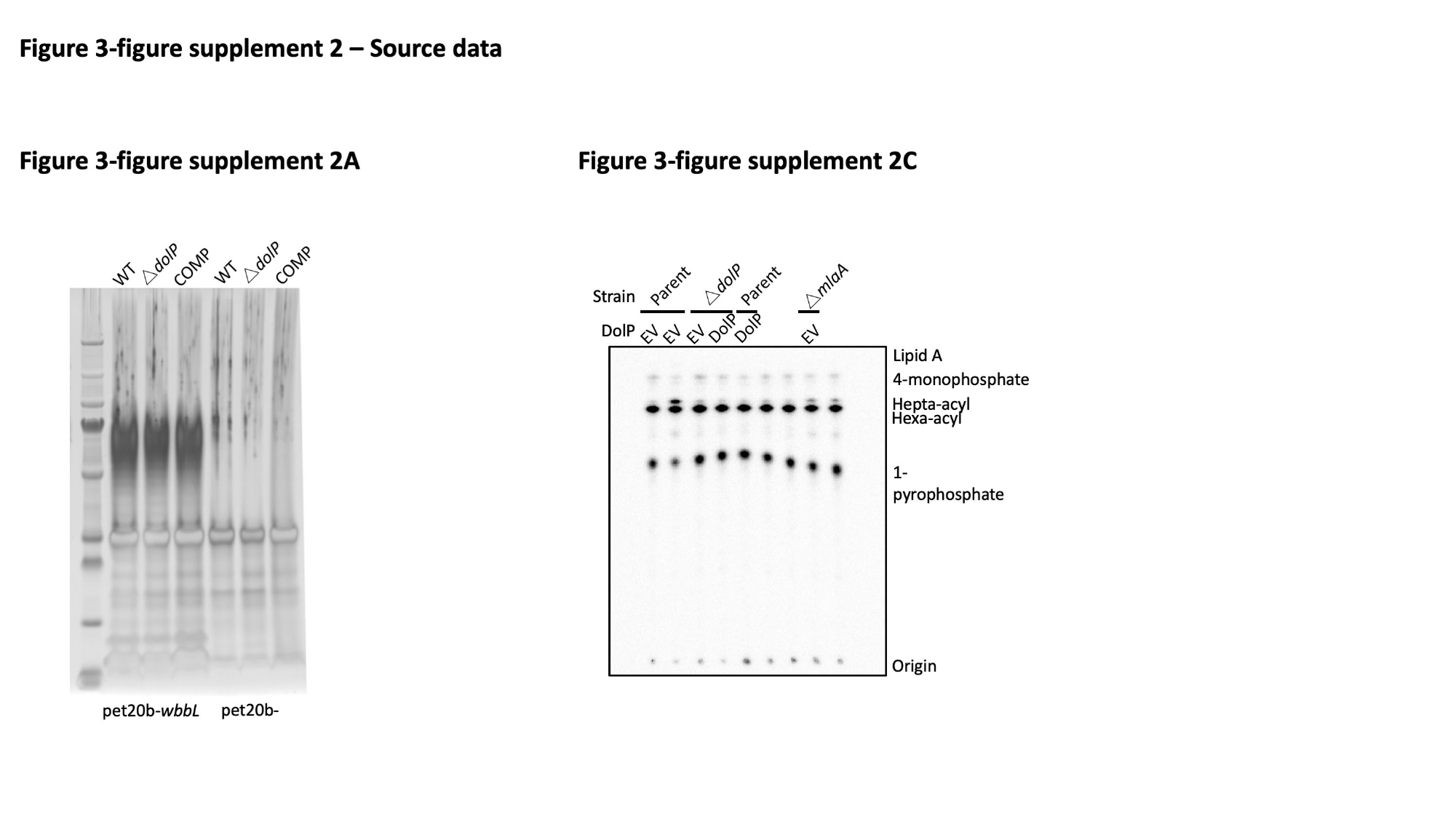

Supplement: Figure 3—figure supplement 2—source data 1. [file elife-62614-fig3-figsupp2-data1.pptx]

## Slide 1
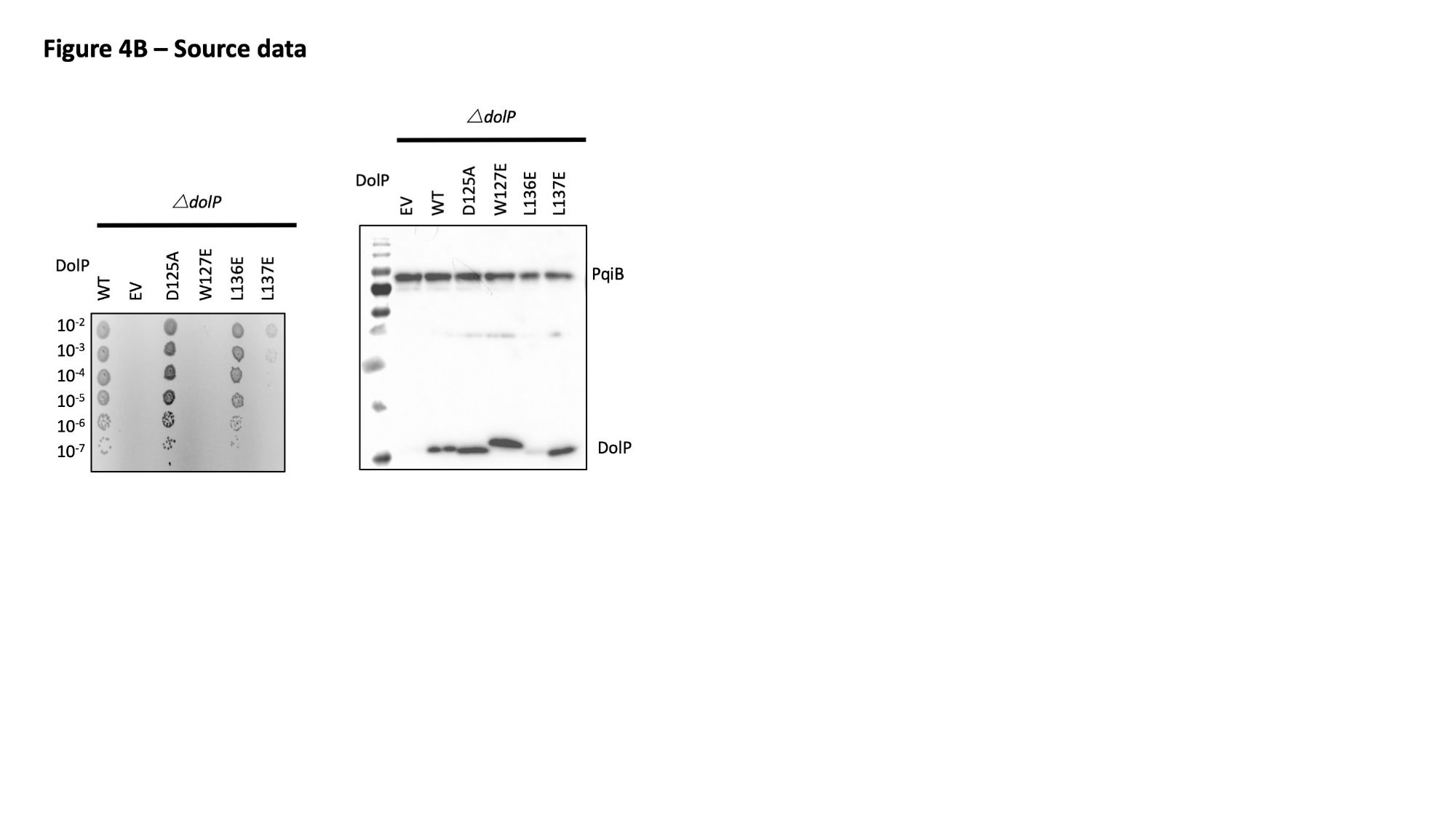

Supplement: Figure 4—source data 1. [file elife-62614-fig4-data1.pptx]

## Slide 1
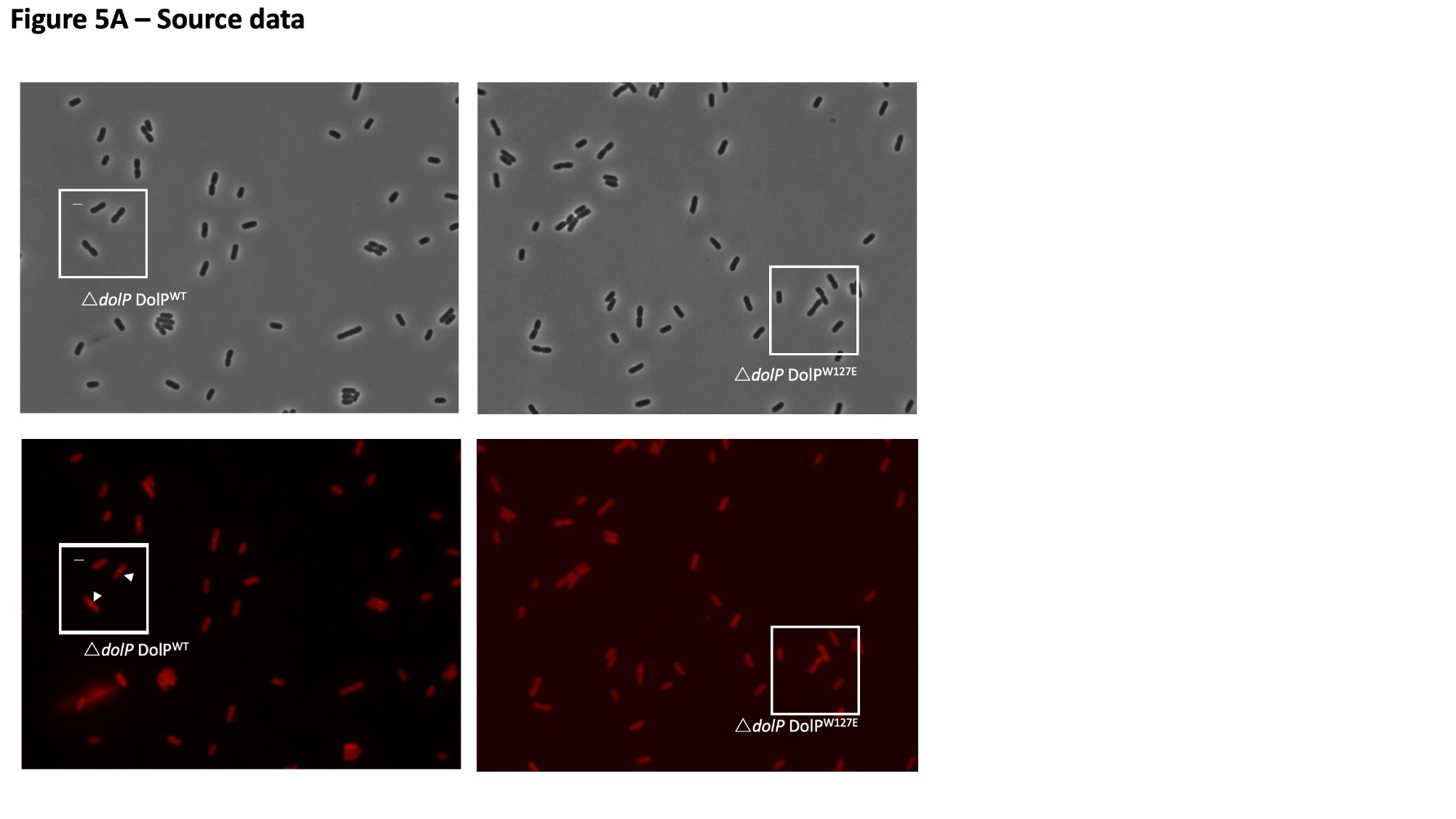

## Slide 2
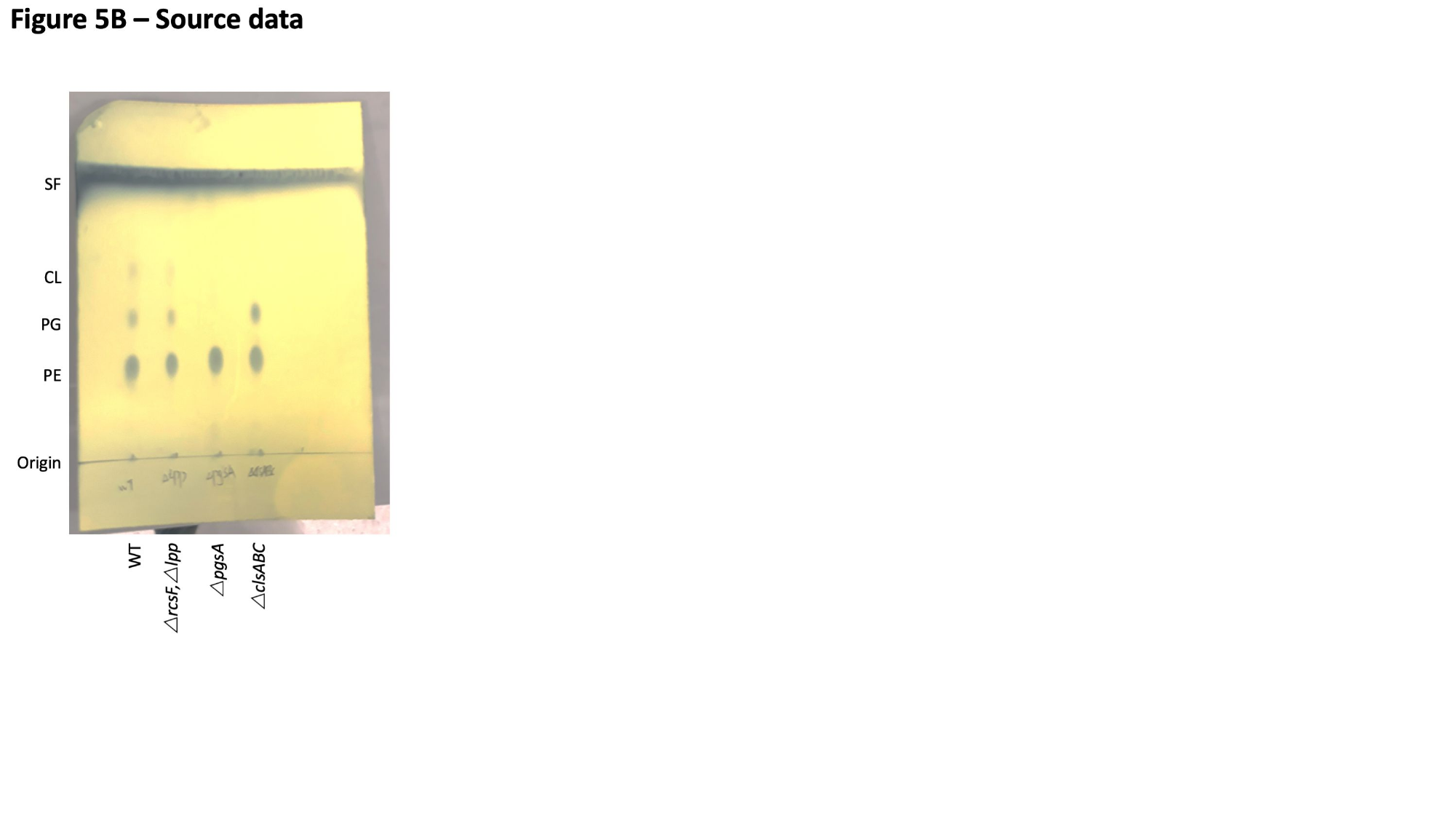

## Slide 3
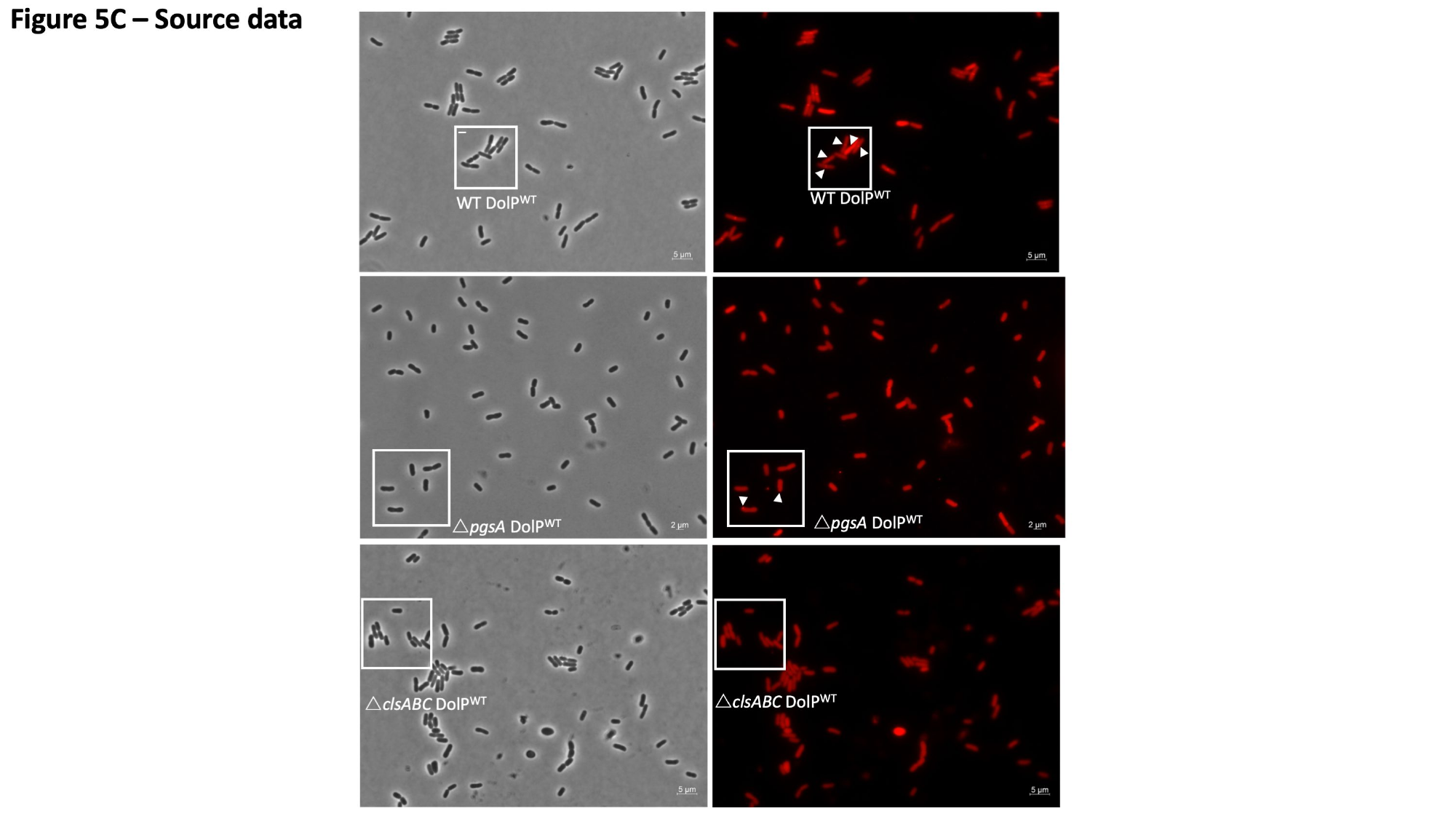

Supplement: Figure 5—source data 1. [file elife-62614-fig5-data1.pptx]
