# Supplementary material for: Structure of dual BON-domain protein DolP identifies phospholipid binding as a new mechanism for protein localisation
Source: eLife. 2020 Dec 14;9:e62614. doi: 10.7554/eLife.62614 (PMC7806268; doi:10.7554/eLife.62614)
Supplement: Transparent reporting form [file elife-62614-transrepform.docx]

***eLife’s* transparent reporting form**

We encourage authors to provide detailed information *within their submission* to facilitate the interpretation and replication of experiments. Authors can upload supporting documentation to indicate the use of appropriate reporting guidelines for health-related research (see [EQUATOR Network](http://www.equator-network.org/%20)), life science research (see the [BioSharing Information Resource](https://biosharing.org/)), or the [ARRIVE guidelines](http://www.plosbiology.org/article/info:doi/10.1371/journal.pbio.1000412) for reporting work involving animal research. Where applicable, authors should refer to any relevant reporting standards documents in this form.

If you have any questions, please consult our Journal Policies and/or contact us: [editorial@elifesciences.org](mailto:editorial@elifesciences.org).

**Sample-size estimation**

- You should state whether an appropriate sample size was computed when the study was being designed
- You should state the statistical method of sample size computation and any required assumptions
- If no explicit power analysis was used, you should describe how you decided what sample (replicate) size (number) to use

Please outline where this information can be found within the submission (e.g., sections or figure legends), or explain why this information doesn’t apply to your submission:

This information does not apply to our submission as this is not a clinical study

**Replicates**

- You should report how often each experiment was performed
- You should include a definition of biological versus technical replication
- The data obtained should be provided and sufficient information should be provided to indicate the number of independent biological and/or technical replicates
- If you encountered any outliers, you should describe how these were handled
- Criteria for exclusion/inclusion of data should be clearly stated
- High-throughput sequence data should be uploaded before submission, with a private link for reviewers provided (these are available from both GEO and ArrayExpress)

Please outline where this information can be found within the submission (e.g., sections or figure legends), or explain why this information doesn’t apply to your submission:

This information is included in the Figure Legends and in the Methods section.

**Statistical reporting**

- Statistical analysis methods should be described and justified
- Raw data should be presented in figures whenever informative to do so (typically when N per group is less than 10)
- For each experiment, you should identify the statistical tests used, exact values of N, definitions of center, methods of multiple test correction, and dispersion and precision measures (e.g., mean, median, SD, SEM, confidence intervals; and, for the major substantive results, a measure of effect size (e.g., Pearson's r, Cohen's d)
- Report exact p-values wherever possible alongside the summary statistics and 95% confidence intervals. These should be reported for all key questions and not only when the p-value is less than 0.05.

Please outline where this information can be found within the submission (e.g., sections or figure legends), or explain why this information doesn’t apply to your submission:

This information is included in the Figure Legends and in the Methods section.

(For large datasets, or papers with a very large number of statistical tests, you may upload a single table file with tests, Ns, etc., with reference to sections in the manuscript.)

**Group allocation**

- Indicate how samples were allocated into experimental groups (in the case of clinical studies, please specify allocation to treatment method); if randomization was used, please also state if restricted randomization was applied
- Indicate if masking was used during group allocation, data collection and/or data analysis

Please outline where this information can be found within the submission (e.g., sections or figure legends), or explain why this information doesn’t apply to your submission:

This information does not apply to our submission as our study is not a clinical study.

**Additional data files (“source data”)**

- We encourage you to upload relevant additional data files, such as numerical data that are represented as a graph in a figure, or as a summary table
- Where provided, these should be in the most useful format, and they can be uploaded as “Source data” files linked to a main figure or table
- Include model definition files including the full list of parameters used
- Include code used for data analysis (e.g., R, MatLab)
- Avoid stating that data files are “available upon request”

Please indicate the figures or tables for which source data files have been provided:

Bioinformatic analyses presented in Figure 1 are described in detail in the Methods section and additional data required for the analysis are supplied in Table 1, Supplementary Table 2, and Supplementary Table 2.

For Mass Spectrometry analysis of OM proteome samples, the method is described in detail in the Methods section and the data are presented in Supplementary Table 2.

For the NMR structural analysis, the methods are described in detail in the Methods section, the validation report for PDB 7A2D is provided, the structure is also uploaded to BMRB (19760), and structural statistics are reported in Table 2.

For the lipid interaction analysis, the Methods are described in detail in the Methods section and the top 200 models were ranked according to their experimental energies and statistics derived from the 20 lowest energy conformers reported in Table 5. The lipid titration data is represented in the figures and the source data is supplied in files “Figure-3-and-4-source-data” and “Figure-3-source-data”.

We have supplied original images for Figure 1-figure supplement 1, Figure 1-figure supplement 5, Figure 2, Figure 3-figure supplement 1A,B, Figure 3-figure supplement 2, Figure 4B and Figure 5A,B,C. We have also supplied raw data for Figure 1-figure supplement 4B, Figure 2B, Figure 3-figure supplement 2B,C and D, and Figure 3.

For all other analyses, information is reported in the Figure Legends, or Methods section.
